# The Rocky Road from Preclinical Findings to Successful Targeted Therapy in Pleural Mesothelioma

**DOI:** 10.3390/ijms232113422

**Published:** 2022-11-03

**Authors:** Juuso Paajanen, Raphael Bueno, Assunta De Rienzo

**Affiliations:** The Thoracic Surgery Oncology Laboratory and The International Mesothelioma Program, Division of Thoracic Surgery and the Lung Center, Brigham and Women’s Hospital, Harvard Medical School, 75 Francis Street, Boston, MA 02115, USA

**Keywords:** pleural mesothelioma, genomics, gene therapy, targeted therapy

## Abstract

Pleural mesothelioma (PM) is a rare and aggressive disease that arises from the mesothelial cells lining the pleural cavity. Approximately 80% of PM patients have a history of asbestos exposure. The long latency period of 20–40 years from the time of asbestos exposure to diagnosis, suggests that multiple somatic genetic alterations are required for the tumorigenesis of PM. The genomic landscape of PM has been characterized by inter- and intratumor heterogeneity associated with the impairment of tumor suppressor genes such as *CDKN2A*, *NF2*, and *BAP1*. Current systemic therapies have shown only limited efficacy, and none is approved for patients with relapsed PM. Advances in understanding of the molecular landscape of PM has facilitated several biomarker-driven clinical trials but so far, no predictive biomarkers for targeted therapies are in clinical use. Recent advances in the PM genetics have provided optimism for successful molecular strategies in the future. Here, we summarize the molecular mechanism underlying PM pathogenesis and review potential therapeutic targets.

## 1. Introduction

Pleural mesothelioma (PM) is an aggressive cancer developed from the mesothelial cells lining the pleural cavity. Previous asbestos exposure is the most common risk factor for PM, accounting approximately 80% of cases [1]. The incidence of PM is globally increasing, while it has remained largely unchanged in the United States in the past decades with approximately 2000 annual cases [2]. The median survival of patients with PM is 5–14 months [3], but a subset of patients experiences long-term survival [4,5]. PMs are divided into three histological subtypes that are the major prognostic factors: epithelioid with epithelial-shaped cells, sarcomatoid with spindle-shaped cell, and biphasic (or mixed) with a mixture of the two types of cells [6].

Current management is based on surgery, radiotherapy, and systemic therapies [7,8]. Since clinical signs in early disease stages are absent or non-specific, the tumor is often diagnosed in advanced stages [9]. The efficacy of systemic therapies is limited with only a few positive prospective randomized trials in the last decades. Standard first-line chemotherapy, consisting of platinum-antimetabolite, has remained largely unchanged since 2003 [10]. The addition of anti-angiogenic bevacizumab resulted in 2.7-month survival benefit on selected patients when comparing to standard chemotherapy (NCT00651456) [11]. However, its use is limited by toxicity, and its availability varies geographically. A combined immunotherapy of ipilimumab-nivolumab was approved in 2021 as a first-line systemic therapy for PM, based on a phase III randomized trial in which the median overall survival (OS) increased from 14.1 to 18.1 months (NCT02899299) [12]. Unlike prior studies, this result was mainly driven by patients with non-epithelioid histology.

In the last decade, advances in high-throughput techniques, such as next-generation sequencing (NGS), have allowed the characterization of the molecular basis of PM using genomic and transcriptomic analysis [13,14,15]. Because of its ability to reveal a large number of mutations at a relatively low cost, NGS has been used in clinical oncology to advance personalized treatment of cancer. The availability of genomic data has provided a unique opportunity for uncovering new genes involved in prognosis and response to treatments [16]. Recently, these discoveries have provided the basis of multiple biomarker-driven clinical trials in PM [17,18,19,20,21,22,23]. Here, we review the most common molecular alterations and their potential for targeted therapies in PM.

## 2. Common Genetic Alterations Predisposing to PM

### 2.1. Germline Mutations

Pleural mesothelioma is characterized by a latency of 30–50 years from the time of asbestos exposure to diagnosis, suggesting that multiple somatic genetic events are required for mesothelial cell carcinogenic transformation [24]. The observation that PM clustered in certain families with small environmental asbestos/erionite exposure led to the finding of heterozygous germline mutations in the gene encoding the Breast Cancer Gene 1 (*BRCA1)* associated protein-1 (*BAP1*) [25]. Studies using mouse models showed that mice carrying one abnormal copy of *BAP1* treated with low (0.05 mg/weekly) or standard (0.5 mg/weekly) dose of crocidolite asbestos developed significantly more frequently PM compared with the control wildtype carriers [26]. The observation that germline mutations of *BAP1* gene are inherited in an autosomal dominant pattern [25], and associated with increased risk of other malignancies including cutaneous or uveal melanomas, renal cell carcinomas, linked *BAP1* to a tumor predisposition syndrome [27]. Hereditary PM is important to distinguish from sporadic tumors due to several important clinical differences [28]. Germline mutation carriers can develop pleural or peritoneal mesotheliomas often with no or minimal asbestos exposure [29]. Also, for example, mesotheliomas resulting from *BAP1* germline mutations are less aggressive than sporadic tumors with a median survival ranging from 5 to 7 years [30,31]. The underlying reasons of less aggressive behavior of familial PM is largely unknown, but, interestingly, patients with somatic *BAP1* mutations do not share similar survival advantage [32]. Screening of *BAP1* germline mutation in high-risk families has been proposed and several ongoing trials aim to optimize reliable screening and treatment approaches [28,33].

Several other cancer susceptibility mutations have been found in PM. One study on 198 unrelated mesothelioma patients, including 148 (75%) that originated from pleura, 44 (22%) from peritoneum, 3 (2%) from tunica vaginalis, and 3 (2%) from both pleura and peritoneum identified 24 germline mutations in 13 genes in 12% of patients [34]. *BAP1* was the most common mutation, observed in 3% of tested patients, whereas other germline mutations were found in *BRCA1-2*, Checkpoint Kinase 2 (*CHEK2*), Cyclin-Dependent Kinase Inhibitor 2A (*CDKN2A*), Ataxia- Telangiesctasia Mutated (*ATM*), Meiotic Recombination 11 Homolog A (*MRE11A*), Tumor Protein 53 (*TP53*), Muts Homolog 6 (*MSH6*), Transmembrane Protein 127 (*TMEM127*), Succinate Dehydrogenase Complex Flavoprotein Subunit a (*SDHA*), von Hippel-Lindau Tumor Suppressor (*VHL*), Wilms Tumor (*WT1*) genes.

### 2.2. Somatic Mutations

On average, PM tumors contains less than 1 mutations per million bases, lower than in other malignancies associated with external carcinogens [13,35,36]. Karyotypic and comparative genomic hybridization analyses have identified frequent deletions in chromosomes 1p, 3p, 4p, 4q, 6q, 9p, 13q, 14q, 15q, and 22q, whereas gains, less common, involve chromosome arms 1q, 5p, 7p, 8q, and 17q [37]. NGS studies have revealed that aberrations affecting multiple regions of the genome, such as point mutations, minute deletions, and copy number variations, are frequent [13,14,38]. In addition, epigenetic studies have shown that promoter hypermethylation or histone post-translational modifications, leading to altered gene expression, are common events in PM carcinogenesis [39].

In 2010, our group analyzed the whole genome sequence (WGS) from a PM tumor and the matching normal lung tissue using a combination of sequencing-by-synthesis and pyrosequencing methodologies [40]. This study showed that aneuploidy and inter- and intra-chromosomal rearrangements were more numerous than point mutations. Thirty tumor-specific rearrangements were validated by PCR and Sanger sequencing, 15 of which disrupted 17 gene-encoding regions. One large deletion within the Dipeptidyl-Peptidase 10 (*DPP10*) gene resulted in altered truncated fusion transcript in the tumor. Additional analyses of 53 PM tumors showed that 31 (55%) samples expressed DPP10. Patients whose tumors expressed *DPP10* had statistically significant longer overall survival compared with patients whose tumors do not express *DPP10* (22 months vs. 8 months). In addition, 3 point mutations in the coding regions of NK6 Homeobox 2 (*NKX6*-2) and Nuclear Factor Related To KappaB Binding Protein (*NFRKB*), and amplification of several genes, such as Pterin-4 Alpha-Carbinolamine Dehydratase 2 (*PCBD2*) and Dihydrofolate Reductase (*DHFR*), involved in growth factor signalling and nucleotide synthesis, were identified. Another study conducted whole-exome sequencing (WES) from 22 PMs [35]. This study found 517 somatic mutations across 490 mutated genes with a mean of 23 somatic mutations per tumor (range 2∼51), that altered the structure of the corresponding protein. Interestingly, 97% of the mutations were identified in the same tumor. Frequent genetic alterations were found in *BAP1*, *CDKN2A*, Neurofibromatosis type 2 (*NF2*), and Cullin 1 (*CUL1*) in this series. In one PM tumor, Kang et al. [41] identified a genome-wide allelic loss along with eleven high-confidence non-synonymous variants, including SET domain bifurcated histone lysine methyltransferase 1 (*SETDB1*) and *TP53* by performing WES. Additional targeted deep sequencing of *SETDB1* identified gene inactivation in 7 out of 69 additional PMs. In 2016, our group published a comprehensive genomic analysis of 216 PMs [13]. Using WES of 99 PM tumors and target sequencing of 460 genes in 103 additional PM tumors, we identified somatic variants in 2028 genes with frequent mutations in multiple gene families. We found that 52% of nonsynonymous mutations were predicted to have a functional impact. Ten frequently mutated genes (q-score > 0.8) in multiple PM tumors were identified: *BAP1*, *NF2*, and *TP53*, histone methyltransferases SET domain containing 2 histone lysine methyltransferase (*SETD2*), Dead-Box Helicase 3 X-Linked (*DDX3X*), Unc-51 Like Autophagy Activating Kinase 2 (*ULK2*), Ryanodine Receptor (*RYR2*), Cilia and Flagella Associated Protein 45 (*CFAP45*), *SETDB1*, and Dead-Box Helicase 51 (*DDX51*). This work identified also 43 gene fusions in 22 samples, many involving tumor suppressor genes. Recurrent copy loss including genes such as *BAP1*, *NF2*, Cyclin Dependent Kinase Inhibitor 2B (*CDKN2B*), Large tumor suppressor kinase 2 (*LATS2*), Large tumor suppressor kinase 1 (*LATS1*) and *TP53* were also found. Confirming previous findings [14], The Cancer Genome Atlas (TCGA) study reported that the tumor suppressors *BAP1, CDKN2A, NF2, TP53, LATS2*, and *SETD2* were among the most frequently mutated in 74 PMs samples. In this series, three cases with genome-wide loss of heterozygosity (LOH), affecting more than 80% of the genome, were identified. The analysis of an additional series of 80 PM samples found two additional cases, representing a combined prevalence of 3.2% (5/154). All these cases presented inactivating point mutations in *SETDB1*, and 4 out of 5 cases had also mutations in *TP53*. More recently, WGS was performed on 58 PM samples with matched transcriptome sequencing [42]. Whole genome duplication was a common event, occurring in 29% of cases, with a significant association with shorter OS. This work confirmed frequent mutations in the most frequently mutated genes in PM (i.e., *BAP1*, *NF2*, *TP53*, *SETD2*, *LATS2*) and described new candidate driver genes, such as SET Domain Containing 5 (*SETD5*) and Polybromo 1 (*PBRM1*).

**Figure 1:**
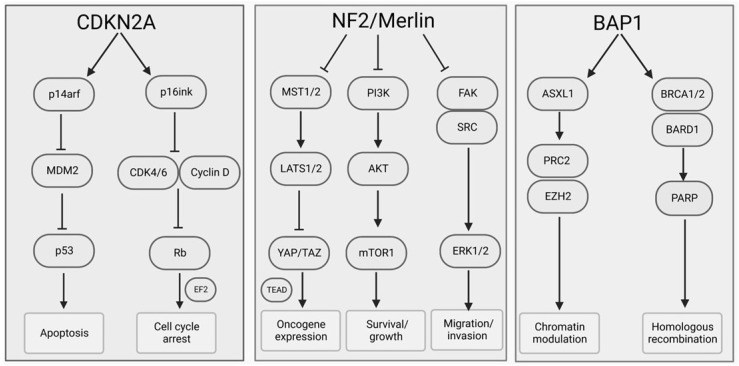
Schematic representation of most frequent tumor suppressor gene pathways related to targeted therapy in mesothelioma. Adapted with permission [43] and created with BioRender.com.

In the era of Precision Medicine, genomic data can direct the treatment for cancer patients. As observed in the past, NGS data indicates that PM development is driven by the inactivation of tumor suppression genes rather than activation of oncogenes. Figure 1 shows schematic illustration of frequently altered genetic pathways in PM.

Therefore, there are considerable challenges in finding specific drugs based on genetic mutations found in patients with PM. Several trials have attempted to target previously described genetic alterations. Table 1 summarizes the genes that are more frequently mutated and their potential therapeutic implications. They will be thoroughly discussed in the following paragraphs.

### 2.3. BAP1

*BAP1* is a tumor suppressor gene that encodes a nuclear deubiquitinase, member of the ubiquitin C-terminal hydrolase family. Most *BAP1* mutations resulted in loss of *BAP1* nuclear localization, where *BAP1* regulates key cellular pathways including gene transcription, cellular differentiation, and DNA repair [28,44,45]. *BAP1* is one of the most commonly mutated genes in PM with over 60% somatic inactivation in sporadic mesotheliomas [14,44]. Immunohistochemical (IHC) nuclear loss has been associated with somatic mutations and it is frequently used in the differential diagnosis of PM [44].

The inactivation of *BAP1* contributes to defects in homologous recombination (HR), caused by failure to deubiquitinate histone H2A on chromatin, leading to accumulation of DNA mutations and chromosomal aberrations (Figure 1) [46]. This genetic instability offers an opportunity for targeting DNA repair factors. Poly ADP-ribose polymerase (PARP) enzymes play a significant role in DNA single-strand repair and base excision pathways. PARP-inhibitors (PARPi) causes accumulation of double-strand breaks leading to synthetic lethality in HR-deficit tumor cells [47]. Currently, PARPis are indicated for the treatment of *BRCA1/2* mutated ovarian, breast, and prostate cancer. Interestingly, in vitro data suggest that PM cells are sensitive to PARPi exposure regardless of *BAP1* status [48,49]. Currently, four early-phase studies using PARPis, rucaparib (NCT03654833), niraparib (NCT03207347, ISRCTN16171129), and olaparib (NCT04515836), are ongoing or recently finished. A phase II single-arm trial evaluated the disease control rate for rucaparib in BAP1/*BRCA1*-deficient relapsed mesothelioma (NCT03654833) [18]. The study screened 36 patients out of which 26 (96% were PMs) were included in the study. Patients received rucaparib 600 mg orally twice daily for six 28-day cycles or until disease progression. The primary endpoint, disease control rate at 12 weeks, was 58% (95% CI 33–77) and at 24 weeks 23% (95% CI 9–44). Disease control was achieved in four patients, and three continued the treatment beyond 12 months. Although all three patients with partial response had *BRCA1* loss, BAP1/*BRCA1* status did not predict treatment response. Another single-arm phase II trial investigated tumor response of olaparib in refractory mesothelioma with or without germline or somatic *BAP1* mutation (NCT03531840) [50]. Twenty-three patients with mesothelioma (16 pleural/7 peritoneal) were recruited. Four patients (1 PM) had germline and 8 (4 PM) had somatic *BAP1* mutations. Of the 16 assessed PM patients, one had (6%) partial response and 11 (69%) had stable disease as a best response. The median progression free survival (PFS) for the whole cohort was 3.6 months (95% CI 2.7–4.2) and OS of 8.7 months (95% CI 4.7-not estimable). Surprisingly, patients with germline, but not somatic *BAP1* mutations had significantly shorter PFS (median 2.3 months, 95% CI 1.3–3.6, versus 4.1 months, 95% CI 2.7–5.5) and OS (median 4.6 months, 95% CI 4.6, versus 9.6 months, 95% CI 5.5-not estimable) than patients with wild-type BAP1. Taken together, these studies suggest that PARPis may have some antitumor efficacy but *BAP1* status cannot be used as biomarker to identify PARPi sensitivity.

Another possible strategy for patients with mutated BAP1 is to target the enhancer or zeste homolog 2 (*EZH2*) histone methyltransferase. *EZH2*, the catalytic subunit of the Polycomb Repressive Complex 2 (PRC2), is an epigenetic regulator of gene expression, which methylates lysine 27 in histone H3 (*H3K27*) (Figure 1). Analysis of the TCGA data [14] revealed that *EZH2* mRNA expression increased in PM samples [51]. In addition, BAP1 knock-out mice resulted in elevated expression of *EZH2*, and EZH2-inhibitors inhibited cell proliferation both in vitro and *in vivo*. A single-arm clinical phase II study recruited patients with BAP1 inactivation determined by IHC (NCT02860286) [21]. The primary endpoint, disease control rate, was evaluated in 61 patients receiving EZH2-inhibitor tazemetostat, including 57 (93%) PMs. The disease control rate was 54% (95% CI 42–67) at 12 weeks and 33% (95% CI 21–45) at 24 weeks in the 61 patients with an overall response rate of 3%. Serious adverse events were reported in 25 (34%) of 74 patients, with no treatment-related deaths. Other EZH2-inhibitors studied in preclinical PM models include 3-Deazanplanocin A (DZNep) and EPZ011989 [51]. Additional studies are needed in PM to determine the potential benefits of EZH2-inhibitors.

### 2.4. CDKN2A

*CDKN2A* is a tumor suppressor gene located on the chromosomal region 9p21 along with *CDKN2B* and Methylthioadenosine phosphorylase (*MTAP*) gene. *CDKN2A* gene encodes two proteins, p^14arf^ and p^16ink4a^, which regulates the cell cycle (Figure 1) [52]. Mice models suggests that inactivation of p^14arf^ and p^16ink4a^ accelerate the asbestos-induced PM tumorigenesis [53]. Homozygous deletions of *CDKN2A* detected by fluorescence in situ hybridization are observed in approximately 60 to 80% of PMs, more common in non-epitheliod PM and associated with significantly decreased survival (median OS 285 vs. 339 days) [54,55]. In addition, approximately 20% of PMs are inactivated by methylation of *CDKN2A* [56].

P^14arf^ is involved in cell cycle regulation by inhibiting *MDM2*, which enables activation of p53. In contrast, the functional loss of p^14arf^ increases MDM2 level and inactivates p53 function, which renders cancer cells to uncontrolled proliferation (Figure 1) [57]. One study evaluated p^14arf^ expression by IHC on 76 chemo-naïve PMs and observed strong positivity in 21% of tumors with a significant association with an aggressive immunophenotype [57]. MDM2 inhibitors have demonstrated promising antitumor activity across a range of preclinical cancer models [58]. A phase I clinical study investigated selective MDM2 inhibitor in multiple advanced wild-type p53 tumors, including a single PM(NCT01723020) [59]. They observed acceptable safety profile with a stable disease in 45 of 68 (66%) patients regardless of *MDM2* amplification or overexpression.

P^16ink4a^ binds and suppresses cyclin-dependent kinase (CDK) 4 and 6, leading to phosphorylation of Rb protein and decelerating G1-S cell cycle transition [60]. In the absence of p^16ink4a^, E2F translocate to the nucleus and allows the transition from G1 phase to S phase of the cell cycle leading to continued proliferation of damaged DNA (Figure 1). Selective CDK4/6 inhibitors, palbociclib, ribociclib, and abemaciclib, have been approved by the US Food and Drug Administration (FDA) for hormone receptor positive, HER2 negative, breast cancer. Recently, a multicenter single-arm phase II trial studied the efficacy of abemaciclib in progressed PM with a loss of p^16ink4A^ expression by IHC(NCT03654833,) [19]. Twenty-six patients were included in the study and treated with 200mg twice-daily abemaciclib. The primary endpoint, disease control rate, was 54% at 12 weeks and 23% at 24 weeks. Four (15%) patients had partial response when evaluating the best overall response within 24 weeks from the start of the treatment. Eight patients (27%) had grade 3 or worse treatment-related side-effects including one (4%) deceased patient. The post-hoc analysis of MTAP IHC revealed the loss of expression in 44% of tumors with a significant association of better clinical outcome compared to MTAP-positive tumors (median change of tumor volume −18% vs. 0%).

In conclusion, targeting downstream products of *CDKN2A,* especially p^16ink4a^, have shown promising antitumor activity, and preclinical models have demonstrated that MDM2 antagonists can overcome intrinsic resistance to CDK-inhibitors [61]. Transcriptomic analysis of TCGA cohort by Jang et al. [62] showed that deletion of *CDKN2A* is associated with primary PD-1 blockade resistance in PM. Among 34 PM patients with *CDKN2A* loss., 74% were classified as anti-PD-1-resistant tumors. In a preclinical mouse model, combination of PD-1 and CDK4/6 inhibitors overcame this resistance resulting in markedly suppressed tumor growth.

### 2.5. MTAP

*MTAP* gene is located on 9p21 locus near to *CDK2NA*. *MTAP* deletions have been reported in a variety of solid tumors, including 67% in PM [55]. *MTAP* encodes the S-methyl-5′-thioadenosine phosphorylase implicated in the polyamine metabolism. MTAP deficiency leads to a dependency on de novo purine synthesis, which can be inhibited by L-alanosine. Preclinical studies have demonstrated selective cytotoxic activity of L-alanosine in variety of MTAP-deficient tumors, whereas similar data in PM is lacking [63,64]. In 2009, phase II trial investigated efficacy and safety of L-alanosine in 55 MTAP-deficient solid tumors, including 16 PMs [23]. In this study, limited clinical activity with no objective responses was observed. However, 13 (24%) patients had stable disease, including 2 patients with mesothelioma. More recently, *MTAP* deletion has been reported to confer sensitivity to inhibition of the protein arginine methyltransferase (PRMT), writer of arginine methylation in histone and non-histone proteins, or deprivation of its substrate s-adenosylmethionine via inhibition of Methionine Adenosyltransferase 2A (*MAT2A*) [65]. Several early-phase trials (NCT03435250, NCT04794699, NCT05245500, NCT05275478) are investigating these potential targeted therapies in advanced solid tumors.

### 2.6. TP53

*TP53* encodes a nuclear transcription factor p53, which is inactivated in most cancers [66]. p53 signaling network regulates transcription and controls cell apoptosis in the presence of DNA damage. P53 signaling can be inactivated by alterations of *CDKN2A.* Alterations in p53 signaling causes PM cells to become sensitive to G2-checkpoint inhibition [67]. A study with 84 PMs (51 epithelioid, 27 biphasic, 6 sarcomatoid) compared molecular findings using NGS-based gene panel and diagnostic IHC [68]. *TP53* gene alterations were identified in 27%, whereas “diffuse” (i.e., over 80% tumor nuclear positivity with 2+ or 3+ intensity) p53 immunostaining was only seen in 7% of tumors. The remaining IHC findings for p53 were wild-type (79%) or null (14%). Overall, p53 IHC was poorly concordant with *TP53* mutational.

The effects of CBP501, a synthetic duodecapeptide that blocks DNA repair at G2-checkpoint, was tested in an open-label randomized phase II trial (NCT00700336) [69]. Sixty-five patients were randomized to pemetrexed/cisplatin chemotherapy with or without CBP501. The addition of CBP501 showed only limited efficacy: the median PFS increased 1.7 months, while the median OS of only 0.5 months. The response rate was higher in the interventional arm: 31% (95% CI 17.0–47.6) with a disease control rate of 69% (95% CI 52.4–83.0) versus 10% (95% CI 1.2–31.7) and 60% (95% CI 36.1–80.9), respectively. Several novel small molecules, such as RITA and nutlin-3, are shown to restore impaired p53 function in PM cell lines, but are yet to be studied in clinical studies [70].

### 2.7. NF2

Germline mutations of *NF2* can cause benign tumors in the skin and nervous system [71]. *NF2* is also frequently inactivated in several malignant tumors, including PMs [14]. Analyses of 211 PMs showed that the *NF2* mutation rate is highest in sarcomatoid subtype [13]. A study with 84 PMs detected *NF2* molecular alterations in 68% of tumors. Similarly, the loss of merlin, a membrane-cytoskeleton scaffolding protein encoded by *NF2*, was observed in 52% of PMs [68]. *NF2,* along with several other mutations, contributes to dysregulation of Hippo pathway, one of the most commonly mutated signaling pathway in PM [13,72]. For example, in the TCGA cohort [14], 20% of PMs had more than one somatic alteration in this pathway.

Merlin contributes to the phosphorylation of the transcription factor Yes-associated protein (YAP), resulting in activation of Hippo pathway. The inactivation of *NF2/*merlin prevents the phosphorylation of YAP resulting in YAP relocation from the cytosol to nucleus where it interacts with TEA domain transcription factors (TEAD). This leads to expression of genes essential for cell cycle regulation (Figure 1) [73]. Preclinical studies have demonstrated that several YAP-signaling suppressors, such as quinacrine, impair migration, proliferation, and invasion of malignant cells, including PM [74,75,76]. However, these promising preclinical results have not progressed to clinical trials yet.

Focal adhesion kinase (FAK), located in cell cytoplasm, is a ubiquitously expressed non-receptor protein tyrosine kinase regulating cell adhesion, proliferation, survival and cancer stem cell renewal (Figure 1) [77]. Experimental evidence suggest that the functional loss of *NF2* in PM contributes to increased FAK expression and tumor cell invasion [78]. Preclinical studies have demonstrated increased sensitivity to FAK inhibitors in merlin-deficient PM cells and tumor xenograft models [77]. A phase I study investigated FAK inhibitor GSK2256098 on multiple advanced solid tumors, including 29 patients with recurrent mesothelioma (NCT01138033). A trend towards longer PFS was observed in merlin-low (median PFS 23.4 weeks, 95% CI 6.0–28.1) compared to merlin-high (median PFS 11.4 weeks, 95% CI 4.3–22.6) mesotheliomas [79]. In 2017, our group investigated the safety and efficacy of FAK inhibitor defactinib as a neoadjuvant agent before surgical resection [80]. Among 30 treated patients, disease control rate was 80% with a partial response of 13%. There was no clinical benefit related to merlin-status in the tumor. The treatment was well tolerated with no alterations in tumor resectability or mortality, when compared with prior series. More recently, a double-blind placebo-controlled randomized phase II trial investigated defactinib in advanced PM(NCT01870609) [20]. The study stratified 344 patients by merlin expression (high vs. low) and randomized participants to receive either maintenance defactinib or placebo after at least four cycles of first-line chemotherapy. Defactinib was given 400 mg twice a day in continuous 21-day cycles until evidence of disease progression. Seven patients (4.0%) in the defactinib group had partial response, as well as 5 (2.9%) in the placebo group. There were no significant improvements in PFS (median 4.1; 95% CI 2.9–5.6 versus 4.0; 95% CI 2.9–4.2 months) or OS (median 12.7; 95% CI 9.1–21 versus 13.6; 95% CI 9.6–21.2 months) when the survival of patients in the defactinib group was compared to that of patients treated with placebo. Similarly, the quality of life as well as pain and dyspnea scores were similar between the two treatment groups. The study failed to confirm an association between merlin expression and defactinib efficacy: the median PFS and OS were 2.8 and 9.0 months in merlin-low vs. 4.5 months and not reached in merlin-high, respectively.

mTOR is a serine/threonine kinase that plays a role by reducing cell proliferation and is frequently mutated in a variety of malignancies [73]. Inactivation of merlin leads to PI3K/mTOR pathway activation (Figure 1) [81]. Preclinical *NF2*-deficient models demonstrated that mTOR inhibitors could increase merlin expression and decrease cell proliferation by enhancing apoptosis [82]. The therapeutic potential of mTOR inhibitor everolimus was tested in a phase II clinical trial in 59 patients with advanced PM after platinum-based chemotherapy (NCT00770120) [83]. Single-agent everolimus was administered 10 mg once a day until disease progression or unacceptable toxicity. The study showed only limited activity with overall response rate of 2%, and disease control rate of 55%. The median PFS was 2.8 months (95% CI 1.8–3.4) and OS 6.3 months (95% CI 4.0–8.0). Unfortunately, the study did not report any data related to merlin/*NF2* status. A different trial (NCT01024946) investigated everolimus specifically in merlin/*NF2*-deficient PM but the results have not been published yet. Recently, several improved mTOR inhibitors have been developed that may enhance clinical benefit in patients with advanced PM [60,84].

### 2.8. LATS2

*LATS2* encodes a serine/threonine kinase involved in a broad array of programs such as cell cycle regulation, cell motility, and differentiation [85]. Loss of *LATS2* has been identified in several different cancer types, including PM [14]. In a cohort of 266 PM samples, mutations of *LATS2* were observed in 5% of the samples, with a higher frequency in non-epithelioid samples and in those without asbestos exposure [86]. Besides mutations of *NF2,* inactivation of *LATS2* leads to YAP overexpression and dysregulation of Hippo pathway [87]. Thus, targeting YAP/Hippo pathway may be a potential strategy in *LATS2*-deficient PM [75].

## 3. Potential Epigenetic Targets

Along with specific genetic alterations, epigenetic modifications, such as DNA methylation or histone acetylation, leads to gene expression alterations. Molecular studies have identified frequent aberrant epigenetic events that provide the rationale for targeted therapy in PM. For example, *SETD2* and *SETDB1* are altered in 8–10% of PMs [13,41]. Preclinical data suggests that histone deacetylases and DNA methyltransferases can induce apoptosis in PM cell lines [39]. Unfortunately, most trials only reached phase I stage due the toxicity and undesired side effects [39]. For example, the dose limiting toxicities for histone deacetylase inhibitors have been nausea, vomiting, fatigue, and anorexia and myelosuppression for DNA methyltransferase inhibitors. One of the most promising trials was a phase II study in which the histone deacetylase inhibitor, valproate, was used in combination with doxorubicin in patients with unresectable PM after platinum-based chemotherapy [88]. Seven out of 45 patients showed partial responses (response rate 16%; 95% CI 3–25%), although two deaths associated with toxicity were observed. A larger phase III, double-blind, randomized, place-controlled trial did not observed survival benefit for the histone deacetylase inhibitor vorinostat [89]. The efficacy of single agent vorinostat was investigated as a second- or third-line therapy in 661 advanced PM patients. Median OS for vorinostat was 30.7 weeks (95% CI 26.7–36.1) and 27.1 weeks (23.1–31.9) for placebo (HR 0.98, 95% CI 0.83–1.17). The PFS was significantly longer in vorinostat group compared to placebo (HR 0.75, 95% CI 0.63–0.88). Vorinostat was well tolerated, with no significant differences in the incidence of serious adverse events between the study groups.

Another emerging epigenetic target is the ubiquitin-like with plant homeodomain and ring finger domains 1 (UHRF1) [90]. UHRF1 is a multidomain protein that primarily recruits DNA methyltransferase to newly synthetized DNA. It is highly expressed in a variety of cancers, leading to regulation of DNA methylation and histone modification [91]. Reardon and colleagues [90] demonstrated that PM cell lines overexpressed *UHRF1* mRNA and protein. Microarray data showed that *UHRF1* was expressed in all (N = 49) PM specimen, whereas it was not expressed in normal pleura samples from patients with benign pleural diseases (N = 11). Expression data from two different cohorts showed that high *UHRF1* levels are associated with worse prognosis. Although there are not available UHRF1 inhibitors, other compounds, such as the DNA-binding antitumor antibiotic mithramycin, may indirectly target UHRF1 [90].

Arginine is an amino acid that plays an important role in signaling pathways and can be either synthetized in the body or absorbed from the diet. The argininosuccinate synthetase 1 (*ASS1*), which makes cancer cells dependent on exogenous supply, may be used for targeted therapy for arginine auxotrophic cancers [92]. Preclinical studies show that the arginine deprivation therapy, pegylated arginine deiminase (ADI-PEG20), is synthetically lethal in *ASS1* deficient tumors with a synergistic effect to antifolate cytotoxicity [93]. The clinical activity of ADI-PEG20 was investigated in a randomized phase II trial in 68 advanced PMs (NCT01279967) [17]. Eligible patients were screened for ASS1 IHC and patients with advanced ASS1-deficient PM, defined by >50% low expressor cells detected by IHC, were included. Patients were randomized to receive a weekly intramuscular injection of ADI-PEG20 up to 6 months or best supportive care. No partial or complete radiological responses were noted. The median PFS in ADI-PEG20 group (3.2 months, IQR 1.8–5.5) was significantly higher than the control group (2.0 months, IQR 1.8–3.6; HR 0.56, 95% CI 0.33–0.96). The median OS did not differ between the study groups (median OS 11.5 months versus 11.1 months; HR 0.68, 95% CI 0.39–1.16). The prespecified subgroup analyses demonstrated greatest benefit for patients with ASS1 loss greater than 75%. Furthermore, a phase I trial found that the combination of ADI-PEG20 and pemetrexed/cisplatin led to disease control rate of 93.5% with a partial response rate of 35.5% in a cohort of 32 PM patients [22]. Based on these findings, a placebo-controlled phase II/III trial is now evaluating the efficacy of ADI-PEG20 combined with chemotherapy in ASS1 deficient non-epithelioid PM patients (NCT02709512).

## 4. Conclusions and Future Perspectives

NGS has revolutionized the study of cancer genetics and led to discovery of several frequently mutated genes in PM. Unlike many other solid tumors, PM is characterized by inactivation of tumor suppressor genes, for which direct pharmacological targeting is challenging. Recently, several preclinical studies and, especially, biomarker-driven clinical trials have shown promising development (Table 2). However, no predictive biomarkers for targeted therapies have been adopted for wide clinical use so far. The inherent inter- and intra-tumor molecular heterogeneity seen in PMs may explain previous challenges in the drug development [94]. Additional studies with novel techniques, such as single-cell and single-nucleus RNA sequencing [95], may enhance new molecular target and mechanisms of resistance identification.

## Figures and Tables

**Table 1:** Common somatic mutations in PM reported by Bueno et al. [11] and their potential therapeutic targets.

Gene Name (Symbol)	Chromosomal Region	Mutation Frequency, (N = 202)	Potential Therapeutic Targets
BRCA1 associated protein 1 (*BAP1*)	3p21.1	35 (17%)	EZH2; PARP
Cyclin-dependent kinase inhibitor 2A (*CDKN2A*)	9p21.3	NA	MDM2; p53; CDK4/6
Methylthioadenosine phosphorylase (*MTAP*)	9p21.3	NA	Adenylosuccinate synthetase; MAT2A; PRMT
Tumor protein P53 (*TP53*)	17p13.1	14 (7%)	G2-checkpoint; MDM2; p53
Neurofibromatosis 2 (*NF2*)	22q12.2	32 (16%)	YAP-TEAD; FAK; mTOR and PI3K
The large tumor suppressor kinase 2 (*LATS2*)	13q12.11	1 (1%)	YAP-TEAD
SET domain containing 2 histone lysine methyltransferase (*SETD2*)	3p21.31	17 (8%)	Histone deacetylase; DNA methyltransferase
SET domain bifurcated histone lysine methyltransferase 1 (*SETDB1*)	1q21	3 (1%)	Histone deacetylase; DNA methyltransferase

**Table 2:** Key clinical phase II trials using genetic stratification in pleural mesothelioma.

Author [Ref]	Design	Stratification	Treatment	Control Group	Subjects	PM ^1^ Patients	ORR ^2^, %	Median PFS ^3^, Months	Median OS ^4^, Months
Kindler [23]	Single-arm	MTAP	L-alanosine	None	Solid tumors	16	0	2.1	5.5
Fennell [19]	Single-arm	P^16ink4A^	Abemaciclib	None	Pretreated advanced PM	26	12	4.2	7.1
Fennell [18]	Single-arm	BAP1/BRCA1	Rucaparib	None	Pretreated advanced PM	25	12	4.1	9.5
Zauderer [21]	Single-arm	BAP1	Tazemetostat	None	Pretreated advanced PM	68	3	4.1	8.3
Fennell [20]	Randomized, double-blind	Merlin	Defactinib	Placebo	Pretreated Advanced PM	344	4.0 vs. 2.9	4.1 vs. 4.0	12.7 vs. 13.6
Szlosarek [17]	Randomized	ASS1	ADI-PEG20	BSC ^5^	Advanced PM	68	0	3.2 vs. 2.0	11.5 vs. 11.1

^1^ PM, pleural mesothelioma; ^2^ ORR, objective response rate; ^3^ PFS, progression-free survival; ^4^ OS, overall survival; ^5^ BSC, best supportive care.

## Data Availability

Not applicable.

## References

[1] Attanoos R., Gibbs A. (1997). Pathology of Malignant Mesothelioma. Histopathology.

[2] Keshava H.B., Tang A., Siddiqui H.U., Raja S., Raymond D.P., Bribriesco A., Stevenson J., Murthy S.C., Ahmad U. (2019). Largely Unchanged Annual Incidence and Overall Survival of Pleural Mesothelioma in the USA. World J. Surg..

[3] Verma V., Ahern C.A., Berlind C.G., Lindsay W.D., Shabason J., Sharma S., Culligan M.J., Grover S., Friedberg J.S., Simone C.B. (2018). Survival by Histologic Subtype of Malignant Pleural Mesothelioma and the Impact of Surgical Resection on Overall Survival. Clin. Lung Cancer.

[4] Paajanen J., Laaksonen S., Kettunen E., Ilonen I., Vehmas T., Salo J., Räsänen J., Sutinen E., Ollila H., Mäyränpää M.I. (2020). Histopathological features of epithelioid malignant pleural mesotheliomas in patients with extended survival. Hum. Pathol..

[5] Paajanen J., Laaksonen S., Ilonen I., Vehmas T., Mäyränpää M.I., Sutinen E., Kettunen E., Salo J.A., Räsänen J., Wolff H. (2020). Clinical Features in Patients With Malignant Pleural Mesothelioma With 5-Year Survival and Evaluation of Original Diagnoses. Clin. Lung Cancer.

[6] Brcic L., Kern I. (2020). Clinical significance of histologic subtyping of malignant pleural mesothelioma. Transl. Lung Cancer Res..

[7] Kindler H.L., Ismaila N., Armato S.G., Bueno R., Hesdorffer M., Jahan T., Jones C.M., Miettinen M., Pass H., Rimner A. (2018). Treatment of malignant pleural mesothelioma: American society of clinical oncology clinical practice guideline. J. Clin. Oncol..

[8] Lapidot M., Gill R.R., Mazzola E., Freyaldenhoven S., Swanson S.J., Jaklitsch M.T., Sugarbaker D.J., Bueno R. (2020). Pleurectomy Decortication in the Treatment of Malignant Pleural Mesothelioma. Ann. Surg..

[9] Nicolini F., Bocchini M., Bronte G., Delmonte A., Guidoboni M., Crinò L., Mazza M. (2020). Malignant Pleural Mesothelioma: State-of-the-Art on Current Therapies and Promises for the Future. Front. Oncol..

[10] Vogelzang N.J., Rusthoven J.J., Symanowski J., Denham C., Kaukel E., Ruffie P., Gatzemeier U., Boyer M., Emri S., Manegold C. (2003). Phase III Study of Pemetrexed in Combination With Cisplatin Versus Cisplatin Alone in Patients with Malignant Pleural Mesothelioma. J. Clin. Oncol..

[11] Zalcman G., Mazieres J., Margery J., Greillier L., Audigier-Valette C., Moro-Sibilot D., Molinier O., Corre R., Monnet I., Gounant V. (2016). Bevacizumab for newly diagnosed pleural mesothelioma in the Mesothelioma Avastin Cisplatin Pemetrexed Study (MAPS): A randomised, controlled, open-label, phase 3 trial. Lancet.

[12] Baas P., Scherpereel A., Nowak A.K., Fujimoto N., Peters S., Tsao A.S., Mansfield A.S., Popat S., Jahan T., Antonia S. (2021). First-line nivolumab plus ipilimumab in unresectable malignant pleural mesothelioma (CheckMate 743): A multicentre, randomised, open-label, phase 3 trial. Lancet.

[13] Bueno R., Stawiski E.W., Goldstein L.D., Durinck S., De Rienzo A., Modrusan Z., Gnad F., Nguyen T.T., Jaiswal B.S., Chirieac L.R. (2016). Comprehensive genomic analysis of malignant pleural mesothelioma identifies recurrent mutations, gene fusions and splicing alterations. Nat. Genet..

[14] Hmeljak J., Sanchez-Vega F., Hoadley K.A., Shih J., Stewart C., Heiman D., Tarpey P., Danilova L., Drill E., Gibb E.A. (2018). Integrative molecular characterization of malignant pleural mesothelioma. Cancer Discov..

[15] De Reynies A., Jaurand M.C., Renier A., Couchy G., Hysi I., Elarouci N., Galateau-Sallé F., Copin M.C., Hofman P., Cazes A. (2014). Molecular classification of malignant pleural mesothelioma: Identification of a poor prognosis subgroup linked to the epithelial-to-mesenchymal transition. Clin. Cancer Res..

[16] Severson D.T., De Rienzo A., Bueno R. (2020). Mesothelioma in the age of “Omics”: Before and after The Cancer Genome Atlas. J. Thorac. Cardiovasc. Surg..

[17] Szlosarek P.W., Steele J.P., Nolan L., Gilligan D., Taylor P., Spicer J., Lind M., Mitra S., Shamash J., Phillips M.M. (2017). Arginine Deprivation With Pegylated Arginine Deiminase in Patients With Argininosuccinate Synthetase 1–Deficient Malignant Pleural Mesothelioma: A Randomized Clinical Trial. JAMA Oncol..

[18] Fennell D.A., King A., Mohammed S., Branson A., Brookes C., Darlison L., Dawson A.G., Gaba A., Hutka M., Morgan B. (2021). Rucaparib in patients with BAP1-deficient or BRCA1-deficient mesothelioma (MiST1): An open-label, single-arm, phase 2a clinical trial. Lancet. Respir. Med..

[19] Fennell D.A., King A., Mohammed S., Greystoke A., Anthony S., Poile C., Nusrat N., Scotland M., Bhundia V., Branson A. (2022). Abemaciclib in patients with p16ink4A-deficient mesothelioma (MiST2): A single-arm, open-label, phase 2 trial. Lancet Oncol..

[20] Fennell D.A., Baas P., Taylor P., Nowak A.K., Gilligan D., Nakano T., Pachter J.A., Weaver D.T., Scherpereel A., Pavlakis N. (2019). Maintenance Defactinib Versus Placebo After First-Line Chemotherapy in Patients With Merlin-Stratified Pleural Mesothelioma: COMMAND—A Double-Blind, Randomized, Phase II Study. J. Clin. Oncol..

[21] Zauderer M.G., Szlosarek P.W., Le Moulec S., Popat S., Taylor P., Planchard D., Scherpereel A., Koczywas M., Forster M., Cameron R.B. (2022). EZH2 inhibitor tazemetostat in patients with relapsed or refractory, BAP1-inactivated malignant pleural mesothelioma: A multicentre, open-label, phase 2 study. Lancet Oncol..

[22] Szlosarek P.W., Phillips M.M., Pavlyk I., Steele J., Shamash J., Spicer J., Kumar S., Pacey S., Feng X., Johnston A. (2020). Expansion Phase 1 Study of Pegargiminase Plus Pemetrexed and Cisplatin in Patients With Argininosuccinate Synthetase 1–Deficient Mesothelioma: Safety, Efficacy, and Resistance Mechanisms. JTO Clin. Res. Rep..

[23] Kindler H.L., Burris H.A., Sandler A.B., Oliff I.A. (2009). A phase II multicenter study of L-alanosine, a potent inhibitor of adenine biosynthesis, in patients with MTAP-deficient cancer. Investig. New Drugs.

[24] Sekido Y. (2013). Molecular pathogenesis of malignant mesothelioma. Carcinogenesis.

[25] Testa J.R., Cheung M., Pei J., Below J.E., Tan Y., Sementino E., Cox N.J., Dogan A.U., Pass H.I., Trusa S. (2011). Germline BAP1 mutations predispose to malignant mesothelioma. Nat. Genet..

[26] Napolitano A., Pellegrini L., Dey A., Larson D., Tanji M., Flores E.G., Kendrick B., Lapid D., Powers A., Kanodia S. (2016). Minimal asbestos exposure in germline BAP1 heterozygous mice is associated with deregulated inflammatory response and increased risk of mesothelioma. Oncogene.

[27] Carbone M., Ferris L.K., Baumann F., Napolitano A., Lum C.A., Flores E.G., Gaudino G., Powers A., Bryant-Greenwood P., Krausz T. (2012). BAP1 cancer syndrome: Malignant mesothelioma, uveal and cutaneous melanoma, and MBAITs. J. Transl. Med..

[28] Carbone M., Pass H.I., Ak G., Alexander H.R., Baas P., Baumann F., Blakely A.M., Bueno R., Bzura A., Cardillo G. (2022). Medical and surgical care of mesothelioma patients and their relatives carrying germline BAP1 mutations. J. Thorac. Oncol..

[29] Chau C., van Doorn R., van Poppelen N.M., van der Stoep N., Mensenkamp A.R., Sijmons R.H., van Paassen B.W., van den Ouweland A.M.W., Naus N.C., van der Hout A.H. (2019). Families with BAP1-Tumor Predisposition Syndrome in The Netherlands: Path to Identification and a Proposal for Genetic Screening Guidelines. Cancers.

[30] Pastorino S., Yoshikawa Y., Pass H.I., Emi M., Nasu M., Pagano I., Takinishi Y., Yamamoto R., Minaai M., Hashimoto-Tamaoki T. (2018). A Subset of Mesotheliomas With Improved Survival Occurring in Carriers of BAP1 and Other Germline Mutations. J. Clin. Oncol..

[31] Baumann F., Flores E., Napolitano A., Kanodia S., Taioli E., Pass H., Yang H., Carbone M. (2015). Mesothelioma patients with germline BAP1 mutations have 7-fold improved long-term survival. Carcinogenesis.

[32] Zauderer M.G., Bott M., McMillan R., Sima C.S., Rusch V., Krug L.M., Ladanyi M. (2013). Clinical characteristics of patients with malignant pleural mesothelioma harboring somatic BAP1 mutations. J. Thorac. Oncol..

[33] Carbone M., Kanodia S., Chao A., Miller A., Wali A., Weissman D., Adjei A., Baumann F., Boffetta P., Buck B. (2016). Consensus Report of the 2015 Weinman International Conference on Mesothelioma. J. Thorac. Oncol..

[34] Panou V., Gadiraju M., Wolin A., Weipert C.M., Skarda E., Husain A.N., Patel J.D., Rose B., Zhang S.R., Weatherly M. (2018). Frequency of Germline Mutations in Cancer Susceptibility Genes in Malignant Mesothelioma. J. Clin. Oncol..

[35] Guo G., Chmielecki J., Goparaju C., Heguy A., Dolgalev I., Carbone M., Seepo S., Meyerson M., Pass H.I. (2015). Whole-exome sequencing reveals frequent genetic alterations in BAP1, NF2, CDKN2A, and CUL1 in malignant pleural mesothelioma. Cancer Res..

[36] Zhang M., Luo J.-L., Sun Q., Harber J., Dawson A.G., Nakas A., Busacca S., Sharkey A.J., Waller D., Sheaff M.T. (2021). Clonal architecture in mesothelioma is prognostic and shapes the tumour microenvironment. Nat. Commun..

[37] Jean D., Daubriac J., Le Pimpec-Barthes F., Galateau-Salle F., Jaurand M.C. (2012). Molecular changes in mesothelioma with an impact on prognosis and treatment. Arch. Pathol. Lab. Med..

[38] Mansfield A.S., Peikert T., Smadbeck J.B., Udell J.B.M., Garcia-Rivera E., Elsbernd L., Erskine C.L., Van Keulen V.P., Kosari F., Murphy S.J. (2019). Neoantigenic Potential of Complex Chromosomal Rearrangements in Mesothelioma. J. Thorac. Oncol..

[39] Vandermeers F., Neelature Sriramareddy S., Costa C., Hubaux R., Cosse J.-P., Willems L. (2013). The role of epigenetics in malignant pleural mesothelioma. Lung Cancer.

[40] Bueno R., De Rienzo A., Dong L., Gordon G.J., Hercus C.F., Richards W.G., Jensen R.V., Anwar A., Maulik G., Chirieac L.R. (2010). Second Generation Sequencing of the Mesothelioma Tumor Genome. PLoS ONE.

[41] Kang H.C., Kim H.K., Lee S., Mendez P., Kim J.W., Woodard G., Yoon J., Jen K., Fang L.T., Jones K. (2016). Whole exome and targeted deep sequencing identify genome-wide allelic loss and frequent SETDB1 mutations in malignant pleural mesotheliomas. Oncotarget.

[42] Creaney J., Patch A.-M., Addala V., Sneddon S.A., Nones K., Dick I.M., Lee Y.C.G., Newell F., Rouse E.J., Naeini M.M. (2022). Comprehensive genomic and tumour immune profiling reveals potential therapeutic targets in malignant pleural mesothelioma. Genome Med..

[43] Xu D., Yang H., Schmid R., Peng R.-W. (2020). Therapeutic Landscape of Malignant Pleural Mesothelioma: Collateral Vulnerabilities and Evolutionary Dependencies in the Spotlight. Front. Oncol..

[44] Nasu M., Emi M., Pastorino S., Tanji M., Powers A., Luk H., Baumann F., Zhang Y.A., Gazdar A., Kanodia S. (2015). High incidence of somatic BAP1 alterations in sporadic malignant mesothelioma. J. Thorac. Oncol..

[45] De Rienzo A., Chirieac L.R., Hung Y.P., Severson D.T., Freyaldenhoven S., Gustafson C.E., Dao N.T., Meyerovitz C.V., Oster M.E., Jensen R.V. (2021). Large-scale analysis of BAP1 expression reveals novel associations with clinical and molecular features of malignant pleural mesothelioma. J. Pathol..

[46] Yu H., Pak H., Hammond-Martel I., Ghram M., Rodrigue A., Daou S., Barbour H., Corbeil L., Hébert J., Drobetsky E. (2014). Tumor suppressor and deubiquitinase BAP1 promotes DNA double-strand break repair. Proc. Natl. Acad. Sci. USA.

[47] Lord C.J., Ashworth A. (2017). PARP inhibitors: Synthetic lethality in the clinic. Science.

[48] Srinivasan G., Sidhu G.S., Williamson E.A., Jaiswal A.S., Najmunnisa N., Wilcoxen K., Jones D., George T.J.J., Hromas R. (2017). Synthetic lethality in malignant pleural mesothelioma with PARP1 inhibition. Cancer Chemother. Pharmacol..

[49] Rathkey D., Khanal M., Murai J., Zhang J., Sengupta M., Jiang Q., Morrow B., Evans C.N., Chari R., Fetsch P. (2020). Sensitivity of Mesothelioma Cells to PARP Inhibitors Is Not Dependent on BAP1 but Is Enhanced by Temozolomide in Cells With High-Schlafen 11 and Low-O6-methylguanine-DNA Methyltransferase Expression. J. Thorac. Oncol..

[50] Ghafoor A., Mian I., Wagner C., Mallory Y., Agra M.G., Morrow B., Wei J.S., Khan J., Thomas A., Sengupta M. (2021). Phase 2 Study of Olaparib in Malignant Mesothelioma and Correlation of Efficacy With Germline or Somatic Mutations in BAP1 Gene. JTO Clin. Res. Rep..

[51] LaFave L.M., Béguelin W., Koche R., Teater M., Spitzer B., Chramiec A., Papalexi E., Keller M.D., Hricik T., Konstantinoff K. (2015). Loss of BAP1 function leads to EZH2-dependent transformation. Nat. Med..

[52] Grard M., Chatelain C., Delaunay T., Pons-tostivint E. (2021). Homozygous Co-Deletion of Type I Interferons and CDKN2A Genes in Thoracic Cancers: Potential Consequences for Therapy. Front. Oncol..

[53] Altomare D.A., Menges C.W., Xu J., Pei J., Zhang L., Tadevosyan A., Neumann-Domer E., Liu Z., Carbone M., Chudoba I. (2011). Losses of both products of the Cdkn2a/Arf locus contribute to asbestos-induced mesothelioma development and cooperate to accelerate tumorigenesis. PLoS ONE.

[54] Marshall K., Jackson S., Jones J., Holme J., Lyons J., Barrett E., Taylor P., Bishop P., Hodgson C., Green M. (2020). Homozygous deletion of CDKN2A in malignant mesothelioma: Diagnostic utility, patient characteristics and survival in a UK mesothelioma centre. Lung Cancer.

[55] Illei P.B., Rusch V.W., Zakowski M.F., Ladanyi M. (2003). Homozygous deletion of CDKN2A and codeletion of the methylthioadenosine phosphorylase gene in the majority of pleural mesotheliomas. Clin. cancer Res. Off. J. Am. Assoc. Cancer Res..

[56] Wong L., Zhou J., Anderson D., Kratzke R.A. (2002). Inactivation of p16INK4a expression in malignant mesothelioma by methylation. Lung Cancer.

[57] Pezzuto F., Lunardi F., Vedovelli L., Fortarezza F., Urso L., Grosso F., Ceresoli G.L., Kern I., Vlacic G., Faccioli E. (2021). P14/ARF-Positive Malignant Pleural Mesothelioma: A Phenotype With Distinct Immune Microenvironment. Front. Oncol..

[58] Canon J., Osgood T., Olson S.H., Saiki A.Y., Robertson R., Yu D., Eksterowicz J., Ye Q., Jin L., Chen A. (2015). The MDM2 Inhibitor AMG 232 Demonstrates Robust Antitumor Efficacy and Potentiates the Activity of p53-Inducing Cytotoxic Agents. Mol. Cancer Ther..

[59] Gluck W.L., Gounder M.M., Frank R., Eskens F., Blay J.Y., Cassier P.A., Soria J.-C., Chawla S., de Weger V., Wagner A.J. (2020). Phase 1 study of the MDM2 inhibitor AMG 232 in patients with advanced P53 wild-type solid tumors or multiple myeloma. Investig. New Drugs.

[60] Bonelli M., Terenziani R., Zoppi S., Fumarola C., La Monica S., Cretella D., Alfieri R., Cavazzoni A., Digiacomo G., Galetti M. (2020). Dual inhibition of CDK4/6 and PI3K/AKT/mTOR signaling impairs energy metabolism in MPM cancer cells. Int. J. Mol. Sci..

[61] Vilgelm A.E., Saleh N., Shattuck-Brandt R., Riemenschneider K., Slesur L., Chen S.-C., Johnson C.A., Yang J., Blevins A., Yan C. (2019). MDM2 antagonists overcome intrinsic resistance to CDK4/6 inhibition by inducing p21. Sci. Transl. Med..

[62] Jang H., Truong C.Y., Lo E.M., Holmes H.M., Ramos D., Ramineni M., Lee J., Wang D.Y., Pietropaolo M., Ripley R.T. (2022). Inhibition of Cyclin Dependent Kinase 4/6 Overcomes Primary Resistance to Programmed Cell Death 1 Blockade in Malignant Mesothelioma. Ann. Thorac. Surg..

[63] Li W., Su D., Mizobuchi H., Martin D.S., Gu B., Gorlick R., Cole P., Bertino J.R. (2004). Status of methylthioadenosine phosphorylase and its impact on cellular response to L-alanosine and methylmercaptopurine riboside in human soft tissue sarcoma cells. Oncol. Res..

[64] Batova A., Diccianni M.B., Omura-Minamisawa M., Yu J., Carrera C.J., Bridgeman L.J., Kung F.H., Pullen J., Amylon M.D., Yu A.L. (1999). Use of alanosine as a methylthioadenosine phosphorylase-selective therapy for T-cell acute lymphoblastic leukemia in vitro. Cancer Res..

[65] Kalev P., Hyer M.L., Gross S., Konteatis Z., Chen C.-C., Fletcher M., Lein M., Aguado-Fraile E., Frank V., Barnett A. (2021). MAT2A Inhibition Blocks the Growth of MTAP-Deleted Cancer Cells by Reducing PRMT5-Dependent mRNA Splicing and Inducing DNA Damage. Cancer Cell.

[66] Ozaki T., Nakagawara A. (2011). Role of p53 in Cell Death and Human Cancers. Cancers.

[67] Xu D., Liang S.-Q., Yang H., Bruggmann R., Berezowska S., Yang Z., Marti T.M., Hall S.R.R., Gao Y., Kocher G.J. (2020). CRISPR Screening Identifies WEE1 as a Combination Target for Standard Chemotherapy in Malignant Pleural Mesothelioma. Mol. Cancer Ther..

[68] Chapel D.B., Hornick J.L., Barlow J., Bueno R., Sholl L.M. (2022). Clinical and molecular validation of BAP1, MTAP, P53, and Merlin immunohistochemistry in diagnosis of pleural mesothelioma. Mod. Pathol..

[69] Krug L.M., Wozniak A.J., Kindler H.L., Feld R., Koczywas M., Morero J.L., Rodriguez C.P., Ross H.J., Bauman J.E., Orlov S.V. (2014). Randomized phase II trial of pemetrexed/cisplatin with or without CBP501 in patients with advanced malignant pleural mesothelioma. Lung Cancer.

[70] Di Marzo D., Forte I.M., Indovina P., Di Gennaro E., Rizzo V., Giorgi F., Mattioli E., Iannuzzi C.A., Budillon A., Giordano A. (2014). Pharmacological targeting of p53 through RITA is an effective antitumoral strategy for malignant pleural mesothelioma. Cell Cycle.

[71] Evans D.G.R. (2009). Neurofibromatosis type 2 (NF2): A clinical and molecular review. Orphanet J. Rare Dis..

[72] Miyanaga A., Masuda M., Tsuta K., Kawasaki K., Nakamura Y., Sakuma T., Asamura H., Gemma A., Yamada T. (2015). Hippo pathway gene mutations in malignant mesothelioma: Revealed by RNA and targeted exon sequencing. J. Thorac. Oncol..

[73] Sato T., Sekido Y. (2018). NF2/merlin inactivation and potential therapeutic targets in mesothelioma. Int. J. Mol. Sci..

[74] Zhang W.-Q., Dai Y.-Y., Hsu P.-C., Wang H., Cheng L., Yang Y.-L., Wang Y.-C., Xu Z.-D., Liu S., Chan G. (2017). Targeting YAP in malignant pleural mesothelioma. J. Cell. Mol. Med..

[75] Woodard G.A., Yang Y., You L., Jablons D.M. (2017). Drug development against the hippo pathway in mesothelioma. Transl. Lung Cancer Res..

[76] Oien D.B., Sarkar Bhattacharya S., Chien J., Molina J., Shridhar V. (2021). Quinacrine Has Preferential Anticancer Effects on Mesothelioma Cells With Inactivating NF2 Mutations. Front. Pharmacol..

[77] Shapiro I.M., Kolev V.N., Vidal C.M., Kadariya Y., Ring J.E., Wright Q., Weaver D.T., Menges C., Padval M., McClatchey A.I. (2014). Merlin Deficiency Predicts FAK Inhibitor Sensitivity: A Synthetic Lethal Relationship. Sci. Transl. Med..

[78] Poulikakos P.I., Xiao G.-H., Gallagher R., Jablonski S., Jhanwar S.C., Testa J.R. (2006). Re-expression of the tumor suppressor NF2/merlin inhibits invasiveness in mesothelioma cells and negatively regulates FAK. Oncogene.

[79] Soria J.C., Gan H.K., Blagden S.P., Plummer R., Arkenau H.T., Ranson M., Evans T.R.J., Zalcman G., Bahleda R., Hollebecque A. (2016). A phase I, pharmacokinetic and pharmacodynamic study of GSK2256098, a focal adhesion kinase inhibitor, in patients with advanced solid tumors. Ann. Oncol..

[80] Bueno R., Gill R.R., Lizotte P.H., Sprott K., Jackman D.M., Barlow J., Sharma S., Yeap B.Y., Chirieac L.R., Lebenthal A. (2017). Effect of FAK inhibitor defactinib on tumor immune changes and tumor reductions in a phase II window of opportunity study in malignant pleural mesothelioma (MPM). J. Clin. Oncol..

[81] López-Lago M.A., Okada T., Murillo M.M., Socci N., Giancotti F.G. (2009). Loss of the tumor suppressor gene NF2, encoding merlin, constitutively activates integrin-dependent mTORC1 signaling. Mol. Cell. Biol..

[82] Li N., Lu X.Y., Shi W.Y., Mao F.J., Yang X.Y., Luo Y.B., Li W. (2019). Combined mTOR/MEK inhibition prevents proliferation and induces apoptosis in NF2-mutant tumors. Eur. Rev. Med. Pharmacol. Sci..

[83] Ou S.-H.I., Moon J., Garland L.L., Mack P.C., Testa J.R., Tsao A.S., Wozniak A.J., Gandara D.R. (2015). SWOG S0722: Phase II Study of mTOR Inhibitor Everolimus (RAD001) in Advanced Malignant Pleural Mesothelioma (MPM). J. Thorac. Oncol..

[84] Rodrik-Outmezguine V.S., Okaniwa M., Yao Z., Novotny C.J., McWhirter C., Banaji A., Won H., Wong W., Berger M., de Stanchina E. (2016). Overcoming mTOR resistance mutations with a new-generation mTOR inhibitor. Nature.

[85] Furth N., Aylon Y. (2017). The LATS1 and LATS2 tumor suppressors: Beyond the Hippo pathway. Cell Death Differ..

[86] Quetel L., Meiller C., Assié J.B., Blum Y., Imbeaud S., Montagne F., Tranchant R., de Wolf J., Caruso S., Copin M.C. (2020). Genetic alterations of malignant pleural mesothelioma: Association with tumor heterogeneity and overall survival. Mol. Oncol..

[87] Mizuno T., Murakami H., Fujii M., Ishiguro F., Tanaka I., Kondo Y., Akatsuka S., Toyokuni S., Yokoi K., Osada H. (2012). YAP induces malignant mesothelioma cell proliferation by upregulating transcription of cell cycle-promoting genes. Oncogene.

[88] Scherpereel A., Berghmans T., Lafitte J.J., Colinet B., Richez M., Bonduelle Y., Meert A.P., Dhalluin X., Leclercq N., Paesmans M. (2011). Valproate–doxorubicin: Promising therapy for progressing mesothelioma. A phase II study. Eur. Respir. J..

[89] Krug L.M., Kindler H.L., Calvert H., Manegold C., Tsao A.S., Fennell D., Öhman R., Plummer R., Eberhardt W.E.E., Fukuoka K. (2015). Vorinostat in patients with advanced malignant pleural mesothelioma who have progressed on previous chemotherapy (VANTAGE-014): A phase 3, double-blind, randomised, placebo-controlled trial. Lancet. Oncol..

[90] Reardon E.S., Shukla V., Xi S., Gara S.K., Liu Y., Straughan D., Zhang M., Hong J.A., Payabyab E.C., Kumari A. (2021). UHRF1 Is a Novel Druggable Epigenetic Target in Malignant Pleural Mesothelioma. J. Thorac. Oncol..

[91] Ashraf W., Ibrahim A., Alhosin M., Zaayter L., Ouararhni K., Papin C., Ahmad T., Hamiche A., Mély Y., Bronner C. (2017). The epigenetic integrator UHRF1: On the road to become a universal biomarker for cancer. Oncotarget.

[92] Zou S., Wang X., Liu P., Ke C., Xu S. (2019). Arginine metabolism and deprivation in cancer therapy. Biomed. Pharmacother..

[93] Allen M.D., Luong P., Hudson C., Leyton J., Delage B., Ghazaly E., Cutts R., Yuan M., Syed N., Lo Nigro C. (2014). Prognostic and therapeutic impact of argininosuccinate synthetase 1 control in bladder cancer as monitored longitudinally by PET imaging. Cancer Res..

[94] Blum Y., Meiller C., Quetel L., Elarouci N., Ayadi M., Tashtanbaeva D., Armenoult L., Montagne F., Tranchant R., Renier A. (2019). Dissecting heterogeneity in malignant pleural mesothelioma through histo-molecular gradients for clinical applications. Nat. Commun..

[95] Slyper M., Porter C.B.M., Ashenberg O., Waldman J., Drokhlyansky E., Wakiro I., Smillie C., Smith-Rosario G., Wu J., Dionne D. (2020). A single-cell and single-nucleus RNA-Seq toolbox for fresh and frozen human tumors. Nat. Med..

